# Preparation, Characterization, and Stability Evaluation of Taste-Masking Lacosamide Microparticles

**DOI:** 10.3390/ma12061000

**Published:** 2019-03-26

**Authors:** Chang-Soo Han, Seungsu Kim, Dong-Won Oh, Jeong Yeol Yoon, Eun-Seok Park, Yun-Seok Rhee, Ju-Young Kim, Dae Hwan Shin, Dong-Wook Kim, Chun-Woong Park

**Affiliations:** 1 College of Pharmacy, Chungbuk National University, Cheongju 28160, Korea; hsoo0805@naver.com (C.-S.H.); seungsu.kim@sandoz.com (S.K.); odw0531@gmail.com (D.-W.O.); ilyj777@naver.com (J.Y.Y.); dshin@chungbuk.ac.kr (D.H.S.); 2 School of Pharmacy, Sungkyunkwan University, Suwon 16419, Korea; espark@skku.edu; 3 College of Pharmacy, Research Institute of Pharmaceutical sciences, Gyeongsang National University, Jinju 52828, Korea; ysrhee@gnu.ac.kr; 4 College of Pharmacy, Woosuk University, Wanju-gun 55338, Korea; jykim@woosuk.ac.kr; 5 Department of Pharmaceutical Engineering, Cheongju University, Cheongju 28530, Korea

**Keywords:** pH dependent, encapsulation, taste-masking effectiveness, surface enrichment, recrystallization

## Abstract

Lacosamide (LCM) is a third-generation antiepileptic drug. Selective action of the drug on voltage-gated sodium channels reduces side effects. Oral administration of LCM shows good pharmacokinetic profile. However, the bitter taste of LCM is a barrier to the development of oral formulations. In this study, we aimed to prepare encapsulated LCM microparticles (MPs) for masking its bitter taste. Encapsulated LCM MPs were prepared with Eudragit^®^ E100 (E100), a pH-dependent polymer, by spray drying. Three formulations comprising different ratios of LCM and E100 (3:1, 1:1, and 1:3) were prepared. Physicochemical tests showed that LCM was in an amorphous state in the prepared formulations, and they were not miscible. LCM-E100 (1:3) had a rough surface due to surface enrichment of LCM. Increased E100 ratio in LCM-E100 MPs resulted in better taste-making effectiveness: LCM-E100 (1:1) and LCM-E100 (1:3) showed good taste-masking effectiveness, while LCM-E100 (3:1) could not mask the bitter taste of LCM. Dissolution results of the prepared formulations showed good correlation with taste-masking effectiveness. Nevertheless, high E100 ratio reduced the stability of the prepared formulations. Especially the difference in initial dissolution profile observed for LCM-E100 (1:3) indicated rapid reduction in taste-masking effectiveness and surface recrystallization. Therefore, LCM-E100 formulation in the ratio of 1:1 was selected as the best formulation with good taste-masking effectiveness and stability.

## 1. Introduction

Epilepsy is a chronic neurological disorder of the brain which is recurrent and unpredictable [1], and is one of the most common neurological diseases worldwide. 

Lacosamide (LCM; [R]-2-acetamido-*N*-benzyl-3-methoxypropionamide) is a third-generation antiepileptic drug approved by the Food and Drug Administration in 2008 as an adjunctive therapy for partial onset seizures in patients with epilepsy aged ≥17 years in the United states. The drug modulates voltage-gated sodium channels (VGSC) by selectively enhancing slow inactivation. [2] The classical VGSC blocking agents promote fast inactivation and block recovery from fast inactivation without any effect on slow inactivation [3,4]. The selective property of LCM stabilizes hyperexcitable neuronal membranes, inhibits neuronal firing, and reduces long-term channel availability without affecting other physiological functions such as cognitive deterioration [5]. It has been shown to be effective and well-tolerated among patients in clinical trials [6]. Oral administration is the common route of LCM administration, as it is easy and noninvasive. Post-oral dose of LCM shows good pharmacokinetic properties, namely low protein binding capacity (<15%), minimal cytochrome P450 interaction, low potential for drug-drug interactions, fast rate of absorption, fast maximum plasma concentration (within 1–4 h), and long half-life (13 h). However, LCM has an extremely bitter taste that can decrease patient compliance [2,7]. 

Taste-masking technologies are applied to mask the bitter and unpleasant taste of active pharmaceutical ingredients and drugs. The simplest taste-masking approach is the use of flavoring agents or sweeteners. However, their efficacy is limited in cases of very bitter or highly water-soluble drugs that are administered in high doses. Complexation, coating, or granulation with hydrophilic polymers; melting and liquid extrusion; and ion-exchange resin are more advanced taste-masking techniques [8]. To overcome the bitter taste of LCM, film-coated tablets and syrups have been developed under the brand name VIMPAT^®^ by Schwarz Pharma (Monnheim, Germany). Nevertheless, they have the following limitations: (1) film-coated tablets cannot be ground to powder for the use of elderly patients and those who have difficulty in swallowing, and (2) large amounts of sugar is added in syrups to mask the bitter taste of LCM, and they should be prepared in large quantities. These limitations can be overcome by polymer encapsulation with spray drying. Microencapsulation is the frequently used and the most effective taste-masking method. It has the following advantages: rapid, reproductive, continuous, and one-step process; cost-effective and scalable without revision [9].

Eudragit^®^ E100 (E100) is a cationic copolymer comprising dimethylaminoethyl methacrylate, butyl methacrylate, and methyl methacrylate [10]. It is commonly used in oral and topical formulations, and is regarded as nontoxic and safe [11]. It is soluble in gastric fluid at a pH of up to 5.0 [12]. This property could ensure that active pharmaceutical ingredients (APIs) encapsulated in E100 dissolve in the stomach without undergoing disintegration in saliva in the oral cavity. Conversely, E100 prevents LCM from stimulating the tongue and it is completely dissolved in gastric juice without altering drug absorption in the gastrointestinal tract. Notably, the physicochemical stability of a taste-masked formulation depends on the taste-masking effectiveness, quality, safety, and efficacy [13]. Although these formulations completely mask the bitter taste of API, a taste-masked formulation with low stability can lose the taste-masking within a few days. 

In the present study, we aimed to prepare and evaluate taste-masked LCM-E100 microparticles (MPs). The designed formulations were examined in terms of morphology, physiochemical properties, taste masking effectiveness, and stability. Taste-masking effectiveness was evaluated by using electronic tongue, human taste panel, and dissolution tests. The LCM-E100 MPs were stored at 25 °C and 40% relative humidity (RH) for 5 days after spray drying. The taste masking effectiveness and morphology of stored formulations were evaluated by dissolution test and scanning electron microscopy (SEM).

## 2. Materials and Methods

### 2.1. Materials

Lacosamide was supplied by the Nutra specialties Private Co., Ltd., (Tamil Nadu, India). Eudragit E100 (E100) was obtained from Evonik-Degussa GmbH (Essen, Germany). Methanol, ethanol, and acetonitrile used were of high-performance liquid chromatography (HPLC) grade and purchased from Honeywell Burdick & Jackson (Muskegon, MI, USA). Water was distilled using a Milli-Q reagent water system (Billerica, MA, USA). All other chemicals were of reagent grade.

### 2.2. Preparation of LCM-E100 Microparticles 

The LCM-E100 MPs were prepared using an SD-1000 spray dry (EYELA, Tokyo, Japan). Three formulations with different LCM and E100 ratios (*w*/*w*) were prepared. LCM-E100 in the ratio of 3:1, 1:1, and 1:3 were accurately weighed and dissolved in 80% ethanol using a magnetic stirrer (Misung Scientific Co., Ltd., Seoul, Korea). In addition, LCM and E100 were spray dried individually under the same conditions. The operating conditions of the spray dryer were as follows: aspirator flow percent of 100%; air flow rate of 500–600 L/h; inlet temperature of 90 °C; and pump feeding rate of 10%. A standard 0.7-mm nozzle was used, and the outlet temperature was maintained at approximately 40 ± 5 °C during the entire spray-drying process. 

### 2.3. Characterization of LCM-E100 Microparticles

#### 2.3.1. Morphology and Particle Size Distribution

The LCM-E100 MPs were imaged by SEM (ZEISS-GEMINI LEO 1530; Zeiss, Germany). The samples were placed onto a carbon tape and were then coated with platinum using a Hummer VI sputtering device, up to a thickness of 200 Å. Particle size distribution of the prepared formulations was measured with Mastersizer 3000 (Malvern, UK).

#### 2.3.2. Drug Loading Content and Entrapment Efficiency of LCM-E100 Microparticles

The LCM content in the spray-dried formulations was measured by a validated HPLC method. Analysis was performed using an Ultimate 3000^®^ series HPLC system (Thermo scientific, Waltham, MA, USA). The determination was performed on Inertsil 4.6 mm × 26 cm packing L7 column (GL sciences Tokyo, Japan). The mobile phase comprising 50% water and 25% methanol containing 1.3% trifluoroacetic acid (*v*/*v*) was eluted at a flow rate of 1.0 mL/min. The detection wavelength was set at 258 nm. The column temperature was maintained at 25 °C, and the volume of each injected sample was 20 µL. The drug loading content (%) and entrapment efficiency (%) were calculated using the following equations (Equations (1) and (2), respectively). All measurements were conducted in triplicate.

(1) $$\mathrm{Drug}\;\mathrm{loading}\;\mathrm{content}\;(\%)=\;\frac{\mathrm{Weight}\;\mathrm{of}\;\mathrm{the}\;\mathrm{drug}\;\mathrm{in}\;\mathrm{microparticles}}{\mathrm{Weight}\;\mathrm{of}\;\mathrm{the}\;\mathrm{microparticles}}\;\times 100$$

(2) $$\mathrm{Entrapment}\;\mathrm{efficiency}\;(\%)=\;\frac{\mathrm{Weight}\;\mathrm{of}\;\mathrm{the}\;\mathrm{drug}\;\mathrm{in}\;\mathrm{microparticles}}{\mathrm{Weight}\;\mathrm{of}\;\mathrm{the}\;\mathrm{feeding}\;\mathrm{drugs}}\;\times 100$$

#### 2.3.3. Differential Scanning Calorimetry

The thermal behavior and phase transition of LCM-E100 MPs were measured using a differential scanning calorimeter (DSC) (Q2000^®^; TA Instruments, New Castle, DE, USA) with TA Universal Analysis of Advantage software v5.2.6. Each sample was placed in DSC aluminum sample pans that were then sealed and heated from 20 to 200 °C at a heating scan rate of 10 °C /min, with a heating rate of 10 °C/min over a temperature range of 20–200 °C.

#### 2.3.4. Powder X-ray Diffraction

The powder X-ray diffraction patterns of the LCM-E100 MPs were measured using an X-ray diffractometer (PXRD; Bruker AXS, Fitchburg, WI, USA). The scanning range of 2θ was from 5° to 60° with a step size of 0.009°/2θ at 25–30 °C with a Cu radiation source (40 kV and 40 mA).

#### 2.3.5. Fourier Transform Infrared Spectroscopy

The infrared spectra were recorded using a Fourier transform infrared spectrophotometer (FT-IR) 4100 (Jasco, Tokyo, Japan) by following the potassium bromide technique. The spectroscopic wavelength range was from 4000 to 650 cm^−1^.

### 2.4. Evaluation of Taste-Masking Effect

#### 2.4.1. Electronic Tongue Measurement

In vitro taste analysis of LCM and prepared LCM-E100 MPs (3:1, 1:1, and 1:3) was performed using an Astree™ e-Tongue system (Alpha MOS, Toulouse, France). Lacosamide and the prepared MPs equivalent to 10 mg of LCM were accurately weighed and dissolved in 100 mL of distilled water with stirring at 500 rpm for 180 s. Each sample solution was measured using seven selective sensors (ZZ, BA, BB, CA, GA, HA, and JB) of Astree™ e-Tongue system eight times every 120 s. The sensor data were analyzed using Alpha Software (Alpha MOS, v12.46). The last three replicates of eight measurements were used in multivariate data analysis through principal component analysis (PCA) mapping [14,15], because they present less variation and high stability owing to the nature of sensors. 

#### 2.4.2. Human Taste Panel

Sensory evaluation of irritation and bitterness of LCM and the prepared formulations was carried out by six healthy volunteers aged 22–50 years. The protocol and experimental designs were approved by the ethical committee of the Chungbuk National University, College of Pharmacy. Mouth cleaning was performed with tooth brush 30 min before the test, and the clean status was maintained by providing only water, without any food. Lacosamide and three formulations were prepared and provided randomly. The results were recorded on a scoring sheet (0: no taste, 1: threshold, 2: slightly bitter, 3: bitter, and 4: remarkably bitter) immediately. Mouth cleaning was performed with a tooth brush at regular intervals.

#### 2.4.3. In Vitro Dissolution Test

The dissolution profile of LCM and prepared LCM-E100 MPs was evaluated using a USP dissolution test apparatus II (Hanson elite 8, Chatsworth, CA, USA). The number of prepared MPs corresponded to 50 mg of LCM. The formulations were suspended in 900 mL of distilled water and in a buffer solution of pH 1.2, separately [16,17]. A paddle speed of 50 rpm and temperature of 37 ± 0.5 °C were maintained during the experiment. At predetermined time intervals (0, 5, 10, 15, 30, 45, 60, and 120 min), 3 mL of sample was collected and passed through a 0.45-μm filter (Whatman, Maidstone, UK). The aliquots were replaced with fresh dissolution medium. The concentration of LCM was analyzed by HPLC. All experiments were performed in triplicate.

### 2.5. Stability

The formulations were stored in an incubator at 25 °C/40% RH for 5 days. The samples were evaluated as described in Section 2.3.1 and Section 2.4.3.

## 3. Results and Discussion

### 3.1. Preparation of the LCM-E100 Microparticles

LCM-E100 (3:1), LCM-E100 (1:1), and LCM-E100 (1:3) MPs were prepared by spray drying. The formulations were white. Table 1 presents the yield, drug loading content, and entrapment efficiency of the prepared formulations. The yield was only around 40% due to the attachment of MPs to the cyclone and loss of small particles in exhaust air flow. The entrapment efficiency of MPs was almost 100%, indicating that LCM and E100 were properly distributed in the droplets.

### 3.2. Characterization of the LCM-E100 Microparticles

#### 3.2.1. Morphology and Particle Size Distribution of the Prepared Formulations

**Figure 1:**
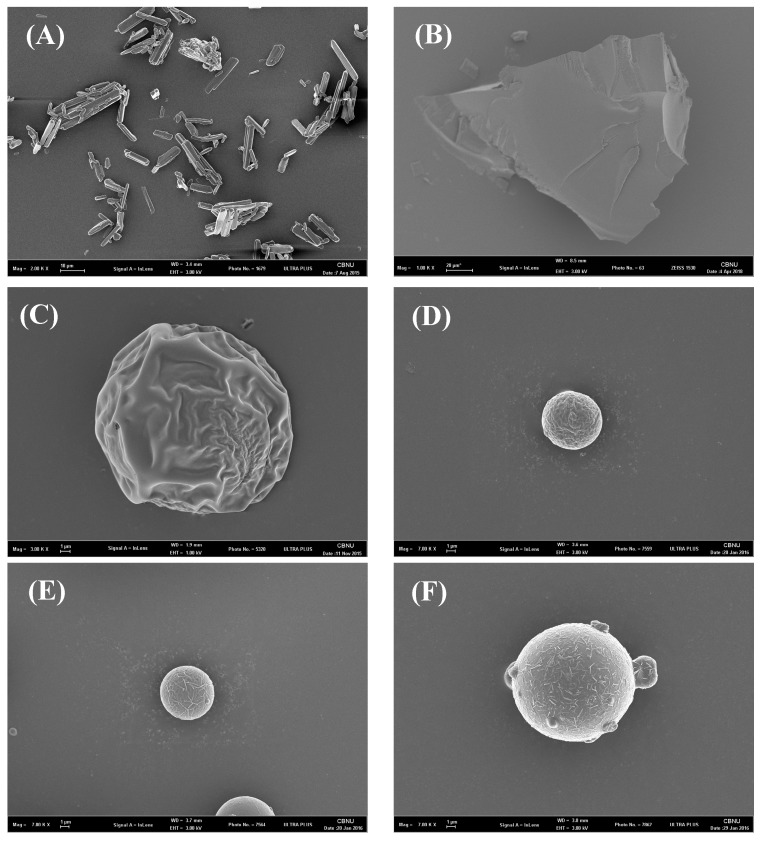
SEM images of (**A**) lacosamide (×2000), (**B**) Eudragit E100 (×1000), (**C**) spray-dried Eudragit E100 (×3000), (**D**) LCM-E100 (3:1) (×7000), (**E**) LCM-E100 (1:1) (×7000), and (**F**) LCM-E100 (1:3) (×7000).

SEM images of the prepared formulations are shown in Figure 1. Lacosamide was rod-shaped and crystalline. E100 was irregularly shaped with a large particle size. Spray-dried E100 had wrinkled surface. LCM-E100 (3:1) and LCM-E100 (1:1) were spherical with smooth surface. LCM-E100 (1:3) was spherical with mild surface roughness. The reason of the difference in shape might be surface enrichment of LCM. Diffusional motion of LCM was faster than the radial velocity of receding droplet surface in the formulation with low polymer ratio. However, it was slower than the radial velocity of receding droplet surface in formulations with high polymer ratio [18]. Therefore, high LCM concentration on the surface of LCM-E100 (1:3) resulted in rough surface. Table 2 presents the particle size distribution of the prepared formulations. The Dv50 of LCM was measured to be 34.5 μm with a broad size range and a size of 800 μm or more. Particle size of > 50 μm has been reported to cause irritation in the mouth [19]. In the present study, the Dv50 of the prepared formulations was < 30 μm with low span value. Thus, they could be administered orally without any irritation and discomfort [20].

#### 3.2.2. PXRD of the Prepared Formulations

**Figure 2:**
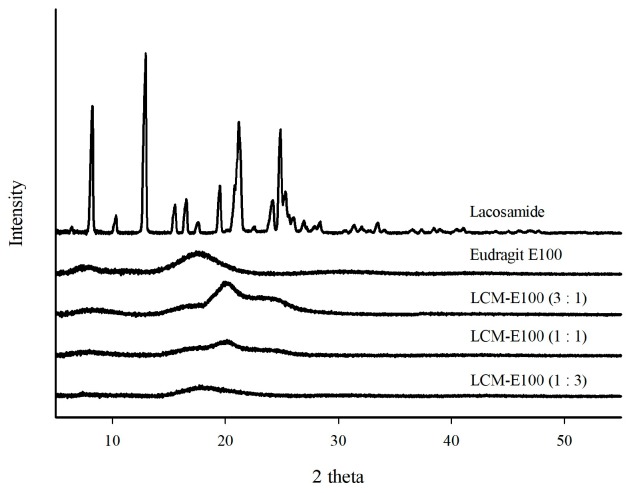
PXRD diffractograms of lacosamide, Eudragit E100, LCM-E100 (3:1), LCM-E100 (1:1) and LCM-E100 (1:3).

The PXRD patterns are shown in Figure 2. The diffractograms of all formulations had no sharp peaks owing to complete phase transformation to an amorphous solid state, whereas raw LCM showed sharp, strong diffraction peaks at 8°, 13°, 21°, and 25°. E100 was observed to be in amorphous state.

#### 3.2.3. DSC of the Prepared Formulations

**Figure 3:**
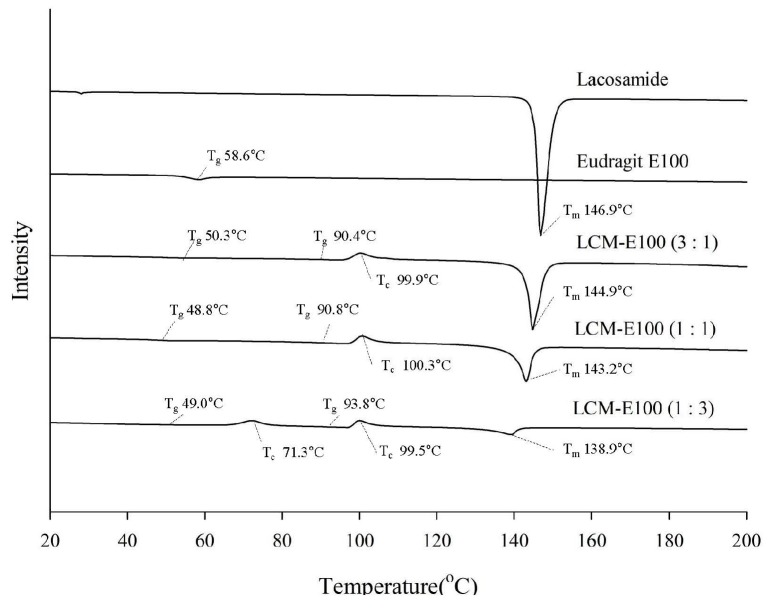
DSC thermograms of lacosamide, Eudragit E100, LCM-E100 (3:1), LCM-E100 (1:1) and LCM-E100 (1:3).

The DSC thermograms are shown in Figure 3. The melting point (*T*_m_) of LCM was confirmed at 146.9 °C with a sharp endothermic peak. E100, which is an amorphous polymer, presented glass transition point (*T*_g_) at 58.6 °C, but Tm peak was not observed. The *T*_g_, crystallization temperature (*T*_c_) and *T*_m_ of the prepared formulations indicated their amorphous state [21]. LCM-E100 (3:1) presented *T*_g_ at 50.3 and 90.4 °C; *T*_c_ at 99.9 °C; and *T*_m_ at 144.9 °C. LCM-E100 (1:1) presented *T*_g_ at 48.8 and 90.8 °C; *T*_c_ at 100.3 °C; and *T*_m_ at 143.2 °C. LCM-E100 (1:3) presented the *T*_g_ at 49.0 and 93.8 °C; *T*_c_ at 71.3 and 99.5 °C; and *T*_m_ at 138.9 °C. The *T*_m_ of all the prepared formulations shifted toward lower temperature than that of LCM. Higher polymer ratio led to shifting of *T*_m_ toward lower temperature. Two *T*_g_ were observed in the prepared formulations, suggesting that LCM and E100 are not miscible in the MPs [22]. If two ingredients are miscible, they show a single *T*_g_ between the *T*_g_ of the pure ingredients, according to Gordon–Taylor equation [23,24]. Specifically, two *T*_c_ (73.5 and 101.8 °C) and two *T*_g_ were observed in LCM-E100 (1:3). This could be because LCM and E100 blended partially [25].

#### 3.2.4. FT-IR of the Prepared Formulations

**Figure 4:**
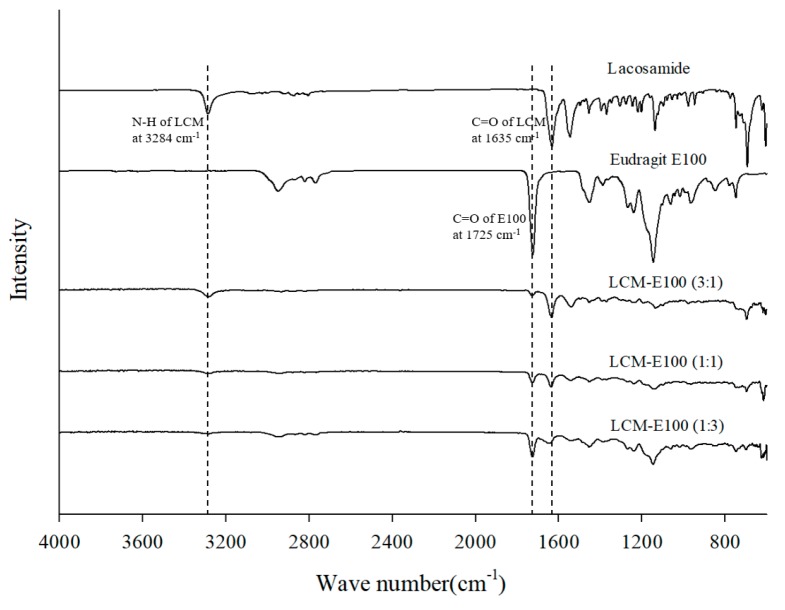
FT-IR spectrums of lacosamide, Eudragit E100, LCM-E100 (3:1), LCM-E100 (1:1) and LCM-E100 (1:3).

The FT-IR spectra are shown in Figure 4. Lacosamide showed the peak of C=O stretching of amide group at 1635 cm^−1^ and peak of N–H stretching of amide group at 3284 cm^−1^. E100 showed the C=O stretching band of ester groups at 1725 cm^−1^. The prepared formulations did not show any new peaks. The intensity of C=O peak of LCM increased in the high LCM ratio formulation. Hydrogen bond was observed in LCM-E100 formulations having hydrogen bond donor and acceptor groups [26]. However, no remarkable shift or increase of bonds in the prepared formulation was observed.

### 3.3. Evaluation of Taste-Masking Effectiveness

#### 3.3.1. Electronic Tongue Test Results of the Prepared Formulations

**Figure 5:**
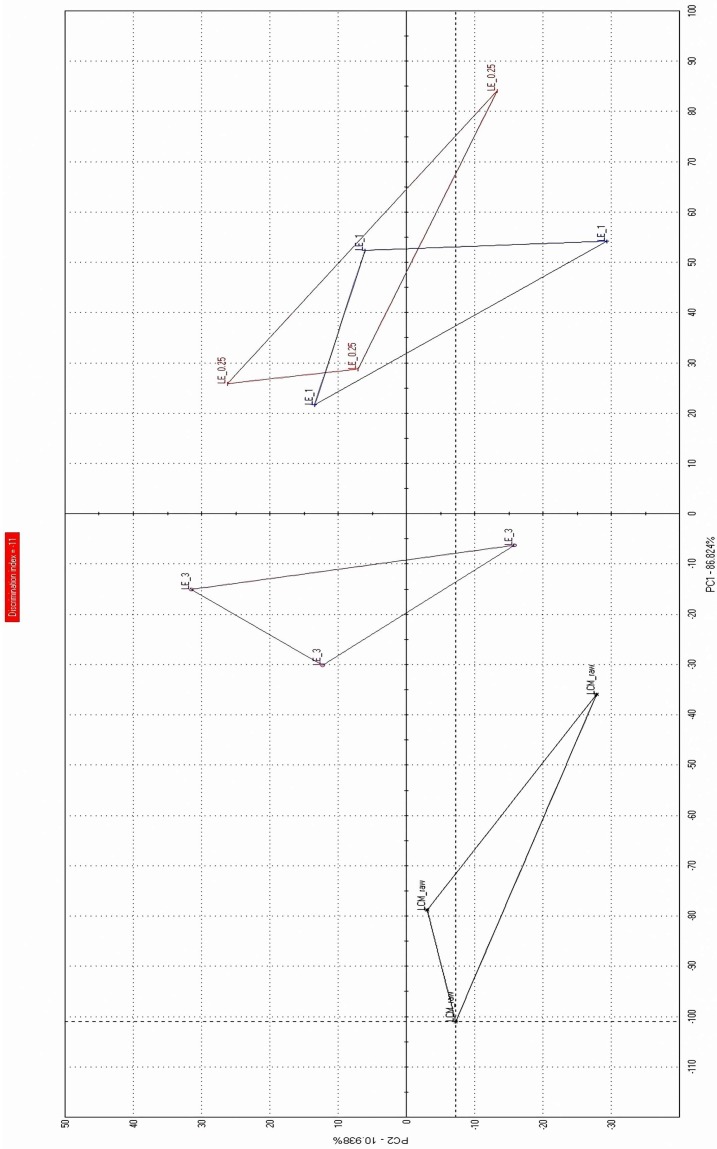
E-tongue test results (PCA map) of lacosamide, LCM-E100 (3:1), LCM-E100 (1:1) and LCM-E100 (1:3).

The PCA map of the e-tongue test is shown in Figure 5. Table 3 presents the distance of the prepared formulations from LCM position in the PCA map. The greater distance from LCM on the PCA map means better taste-masking effectiveness. The taste of the prepared formulations improved when compared with the bitter taste of LCM. LCM-E100 (1:1) and LCM-E100 (1:3) presented good taste-masking effectiveness. The taste of LCM-E100 (3:1) improved when compared with that of LCM, but it was not as effective as the others. These results showed that the MPs with higher polymer ratio had better taste-masking effectiveness. The correlation (*R*^2^) of PCA map distance value of e-tongue and polymer weight ratio showed good relationship (0.94).

#### 3.3.2. Bitterness of the Prepared Formulations

The results of the taste test with human taste panel are presented in Table 3. A higher score indicates bitter taste. Lacosamide was assigned the highest score of 3.67 ± 0.5. Among the six volunteers, four felt that LCM was very bitter. The bitterness of LCM-E100 (3:1) was recorded as 3.17 ± 0.8, and two volunteers felt that the taste was bitter than that of LCM powder. It was considered that the amorphous state of LCM in LCM-E100 (3:1) enhanced instant solubility, and E100 could not prevent contact between the drug and tongue. LCM-E100 (1:1) was assigned a score of 1.83 ± 0.4, and five volunteers reported slightly bitter taste. LCM-E100 (1:3) was assigned a score of 1.17 ± 0.8, which was the lowest score among the prepared formulations. Higher E100 ratio in the prepared formulations resulted in better taste-masking effectiveness. E100 ratio in LCM-E100 and human taste panel presented good correlation (*R*^2^ = 0.98). The difference in the bitterness score of LCM and the prepared formulations was defined by *t*-tests. Significant differences compared with LCM were observed for LCM-E100 (1:1) and LCM-E100 (1:3), as determined by the statistical hypothesis tests (*p* < 0.05). These results suggested that the bitter taste of LCM was masked well in LCM-E100 (1:1) and LCM-E100 (1:3) formulations. 

#### 3.3.3. Dissolution Profile of the Prepared Formulations

**Figure 6:**
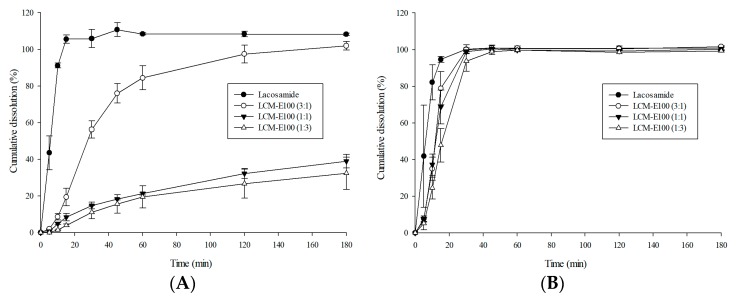
Cumulative dissolution profiles of lacosamide, LCM-E100 (3:1), LCM-E100 (1:1) and LCM-E100 (1:3). (**A**) Dissolution profiles of the prepared formulation at the distilled water, (**B**) Dissolution profiles of the prepared formulation in buffer solution of pH 1.2.

Dissolution profiles of LCM and the prepared formulations are presented in Figure 6. Dissolution test was performed in distilled water and a buffer of pH 1.2. The release profiles in distilled water are shown in Figure 6A. The dissolution of LCM, LCM-E100 (3:1), LCM-E100 (1:1), and LCM-E100 (1:3) was 105.8%, 56.2%, 14.7%, and 11.1%, respectively, at 30 min. Both LCM-E100 (1:1) and LCM-E100 (1:3) showed a remarkable decrease in dissolution when compared with that of LCM, indicating that they can provide enough latency in the mouth. The dissolution rate decreased with increase in polymer ratio and showed good correlation (*R*^2^ = 0.98). The release profiles in the buffer of pH 1.2 are shown in Figure 6B. LCM and the prepared formulations were almost completely released within 30 min. Thus, the prepared formulations are expected to be completely released within 30 min in the gastric juice.

#### 3.3.4. Correlation of The Taste Masking Evaluations

**Figure 7:**
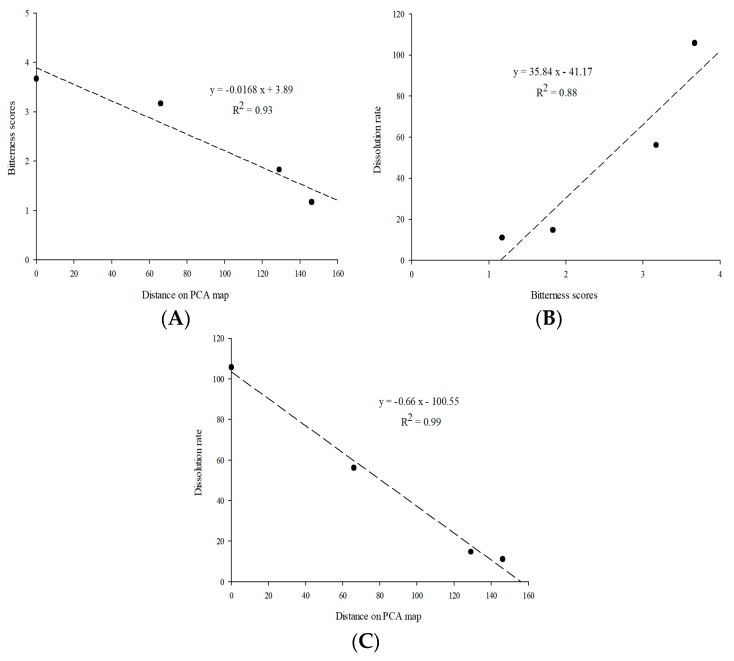
Correlation between results of taste-masking effectiveness. (**A**) correlation between e-tongue test and human taste panel; (**B**) correlation between human taste panel and dissolution profiles at 30 min; and (**C**) correlation between e-tongue test and dissolution profiles at 30 min.

The relationship between the results of taste-mask evaluations is shown in Figure 7. The correlation (*R*^2^) between the e-tongue test and human taste panel test was 0.93. The correlation between dissolution profiles and human taste panel was 0.88, while that between dissolution profile and e-tongue test was 0.99. The bitterness score and dissolution profiles presented a good relationship. The taste-making effectiveness of LCM-E100 MPs could be predicted using dissolution profiles.

### 3.4. Stability of the Prepared Formulations

**Figure 8:**
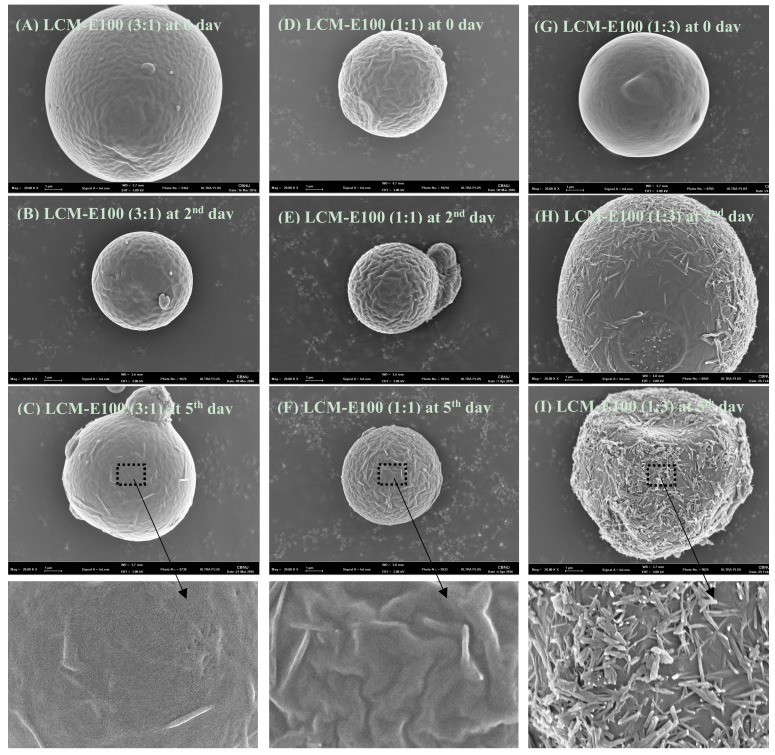
SEM images of the prepared formulations during the stability test. (**A**–**C**) show the SEM images of LCM-E100 (3:1); (**D**–**F**) show the SEM images of LCM-E100 (1:1); (**G**–**I**) show the SEM images of LCM-E100 (1:3) as 0, 2, and 5 days during stability test.

The stability of the prepared formulations was evaluated at 25 °C/40% RH for 5 days. The SEM images of the prepared MPs are shown in Figure 8. LCM-E100 (3:1) and LCM-E100 (1:1) MPs were spherical and had similar surface shape on 0, 2, and 5 days, with no obvious change on their surface. LCM-E100 (1:3) presented loss of spherical shape, shrinkage, and drug recrystallization on surface after 5 days compared with that on 0 day. This was possibly because of the different diffusion rates of the prepared formulations. Lacosamide in LCM-E100 (3:1) and LCM-E100 (1:1) could rapidly diffuse toward the inner side of MPs owing to its small molecular weight in the formulation with low E100 ratio. However, LCM-E100 (1:3) formulation exhibited surface enrichment in an evaporating droplet. This indicated that the diffusion of LCM toward the inner side of MPs was not enough, because E100 dissolved in 80% ethanol was highly evaporated and the movement of LCM toward the surface was faster than its diffusion toward the inner side [27]. The interaction between LCM and E100 was similar to that of hydrogen bonds, preventing recrystallization of LCM in the prepared formulations [28]. In other words, E100 on the surface cannot maintain the amorphous state of LCM in LCM-E100 (1:3) owing to surface enrichment, resulting morphological change in LCM-E100 (1:3) and subsequently affecting the taste-masking effectiveness.

**Figure 9:**
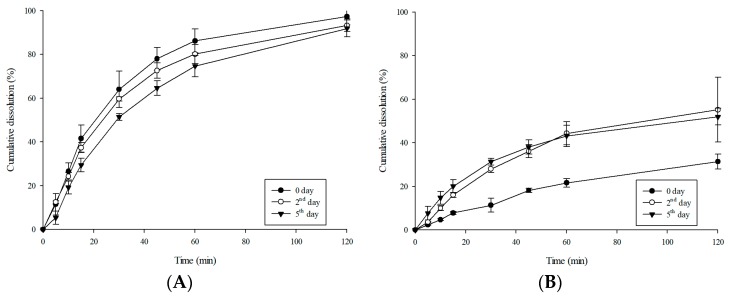

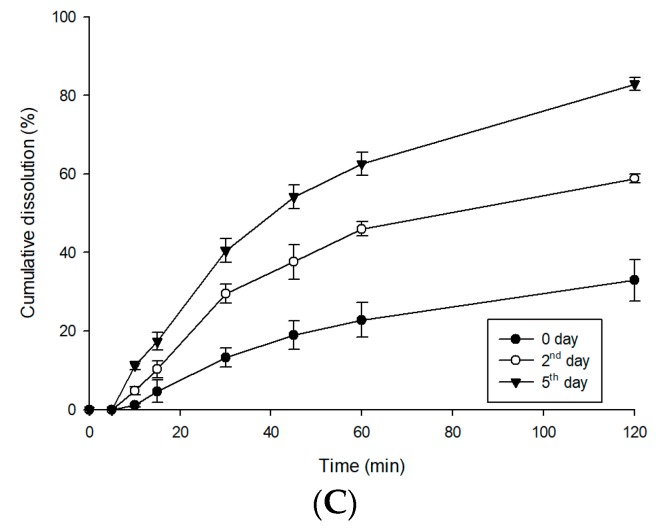
Cumulative dissolution profiles in distilled water during stability test of the prepared formulations. (**A**) Dissolution profiles of LCM-E100 (3:1), (**B**) Dissolution profiles of LCM-E100 (1:1), and (**C**) Dissolution profiles of LCM-E100 (1:3).

The dissolution profiles of the prepared LCM-E100 MPs after 0, 2, and 5 days are shown in Figure 9. The results showed that the difference in dissolution profiles on 0 and 5 days increased with increase in E100 ratio. Specifically, the dissolution rate of LCM-E100 (1:3) changed from 22.9% to 76.3%, with an overall increase of 53.4%. Although LCM-E100 (1:3) had good taste-masking effectiveness at 0 day, it was difficult to maintain the taste-masking effectiveness for 5 days. This might be because of the enhancement of diffusion rate of LCM due to the increased proportion of E100 during the formation of MPs. Furthermore, unequal distribution of LCM in the prepared LCM-E100 MPs increased and the interaction between LCM and E100 decreased. Therefore, LCM-E100 MPs with a high ratio of E100 cannot maintain the amorphous state of LCM during the stability test. Recrystallization of LCM disrupts the E100 barrier in the formulations, affecting the dissolution profile.

## 4. Conclusions

The prepared formulations showed that the drug loading efficiency was nearly 100%. They were spherical with smooth or rough surface depending on the LCM-E100 ratio. The size of the prepared formulations was < 50 μm. Lacosamide in the formulations existed in an amorphous state, and no new chemical interaction was observed. The taste-masking effectiveness of LCM and the prepared MPs was evaluated using e-tongue, human panel and dissolution (in distilled water and a buffer of pH 1.2) test. Increased E100 ratio resulted in LCM-E100 MPs with better taste-masking effectiveness and rapid dissolution at pH 1.2. Regarding stability, high E100 ratio in the formulation resulted in low stability. Furthermore, LCM-E100 (1:3) showed drug recrystallization on the surface and prominent changes in dissolution profiles after 5 days due to surface enrichment. LCM-E100 (1:1) was selected as the best formulation considering its taste-masking effectiveness and physical stability. In this study, evaporation rate was not main factor. Control of the evaporation rate could decrease surface enrichment phenomenon in higher E100 and is expected to make more stable microparticles. The study presents an LCM formulation with improved taste and stability. Taste-masked LCM microparticles can be applicable as a grinded tablet substitute and improve patient adherence in patients who have difficulty in swallowing bitter tablets. 

## Figures and Tables

**Table 1:** Formulation design, yield, drug loading content (%), and entrapment efficiency of the prepared formulations.

Formulations	Drug to Polymer Ratio	Yield (%)	Drug Loading Content (%)	Entrapment Efficiency (%)
LCM-E100 (3:1)	1:0.3	37.2%	73.4% ± 2.2%	97.8% ± 2.9%
LCM-E100 (1:1)	1:1	39.5%	50.6% ± 3.0%	101.2% ± 6.0%
LCM-E100 (1:3)	1:3	38.5%	25.2% ± 1.1%	100.6% ± 4.2%

**Table 2:** Particle size distribution of lacosamide, LCM-E100 (3:1), LCM-E100 (1:1), and LCM-E100 (1:3).

Formulations	Dv10 (μm)	Dv50 (μm)	Dv90 (μm)	Span Value
Lacosamide	3.78	34.5	852	24.59
LCM-E100 (3:1)	4.57	22.1	55.4	2.30
LCM-E100 (1:1)	4.94	17.2	33.7	1.67
LCM-E100 (1:3)	6.60	20.7	34.7	1.35

**Table 3:** Particle size distribution of lacosamide, LCM-E100 (3:1), LCM-E100 (1:1), and LCM-E100 (1:3).

Formulations	E-Tongue Test (Distance on PCA Map)	Human Taste Panel (Bitterness Scores) (*n* = 6, Mean ± S.D.)	Dissolution Profiles at 30 min
Lacosamide	0	3.67 ± 0.5	105.87
LCM-E100 (3:1)	65.97	3.17 ± 0.8	56.23
LCM-E100 (1:1)	128.94	1.83 ± 0.4	14.78
LCM-E100 (1:3)	146.06	1.17 ± 0.8	11.10
Correlation (*R*^2^) (With polymer ratio)	0.94	0.98	0.90
